# Supplementary figures and images for: Multifaceted roles of cGAS-STING pathway in the lung cancer: from mechanisms to translation
Source: PeerJ. 2024 Nov 22;12:e18559. doi: 10.7717/peerj.18559 (PMC11587877; doi:10.7717/peerj.18559)

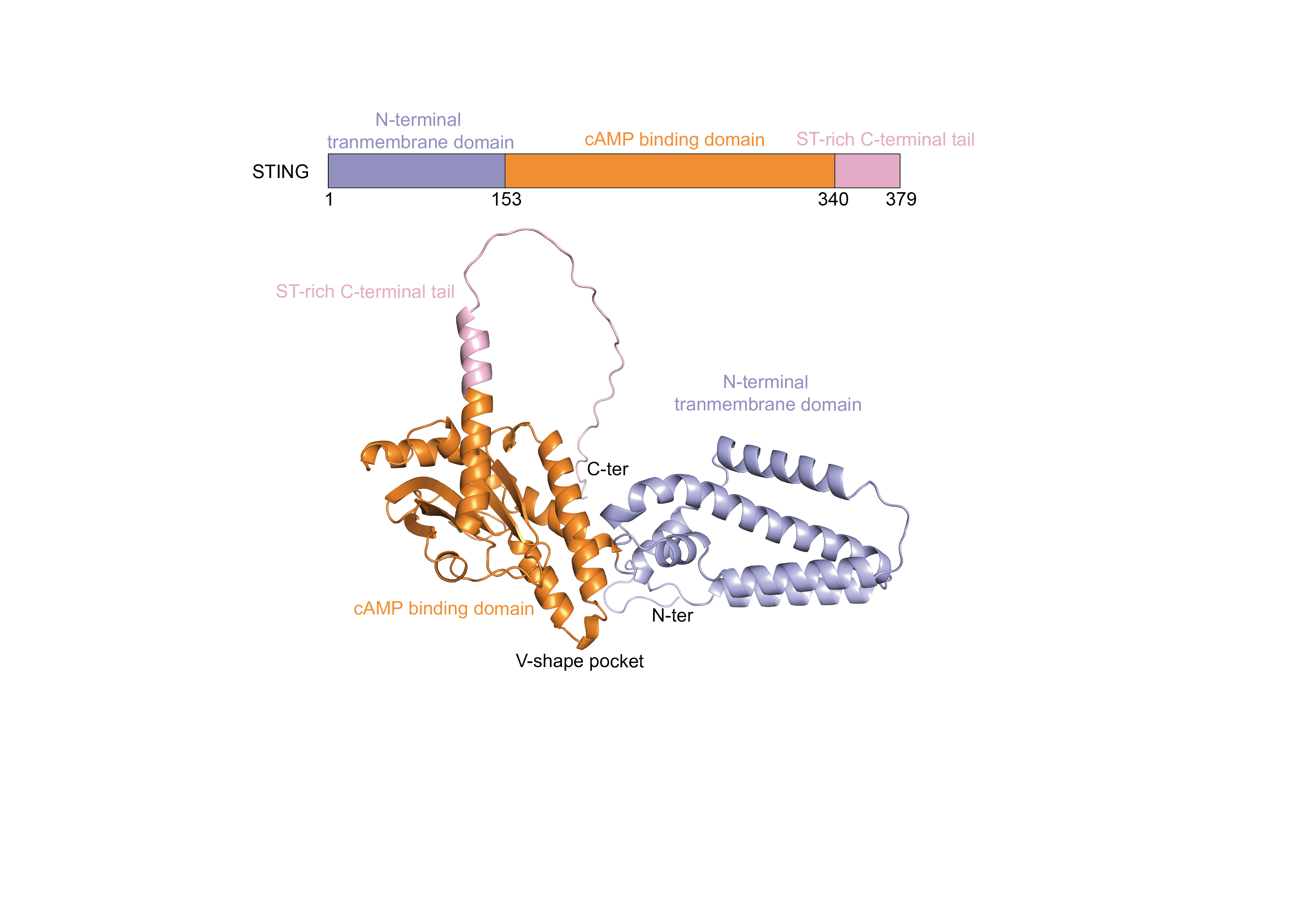

Supplement: Supplemental Information 1 — The human STING structure includes an N-terminal transmembrane region, an intermediate dimerization region, and a C-terminal tail. [file peerj-12-18559-s001.png]
